# Retroperitoneal Mystery: Primary Retroperitoneal Mucinous Cystic Neoplasm With Concomitant Cystic Neoplasm of the Pancreas

**DOI:** 10.1155/criu/3296313

**Published:** 2025-05-22

**Authors:** Luis Gonzalez Miranda, Jessica Qiu, Yubo Wu, Kirsten Greene

**Affiliations:** ^1^Department of Urology, University of Virginia School of Medicine, Charlottesville, Virginia, USA; ^2^University of Virginia School of Medicine, Charlottesville, Virginia, USA; ^3^Department of Pathology, University of Virginia School of Medicine, Charlottesville, Virginia, USA

## Abstract

Urologists are commonly referred patients with retroperitoneal lesions and masses arising from or involving the kidney. In this case, the patient had a retroperitoneal mass identified on imaging which was initially concerning for a cystic renal neoplasm, but it was clearly distinct from all surrounding structures. Pathology found the very uncommon and unexpected diagnosis of a mucinous cystic neoplasm (MCN) with ovarian-type stroma suspected to have arisen from the pancreas. MCNs are lesions that most commonly arise in the ovaries, but less often can arise in extraovarian tissues. Of these extraovarian MCNs, primary retroperitoneal MCNs are exceedingly rare masses with some similarity to their pancreatic and ovarian counterparts. We present a case of an MCN found in the retroperitoneum and initially mistaken for a cystic renal mass, with histological markers and a concomitant pancreatic cyst that suggests possible pancreatic origin. Interestingly, no literature has described pancreatic MCNs without invasive features that have been found in the retroperitoneum without any formal tissue connection to the pancreas. The pathogenesis of retroperitoneal MCNs is still unknown, and as a result, the optimal treatment strategy is unclear.

## 1. Introduction

Solitary retroperitoneal masses can pose a considerable diagnostic challenge to physicians. Masses usually arise from surrounding retroperitoneal organs, and primary nonsarcomatous retroperitoneal masses are exceedingly rare. The differential for primary retroperitoneal cystic masses includes both neoplastic lesions, such as mature teratomas, mucinous cystadenomas, or cystic mesotheliomas, and nonneoplastic lesions like lymphangiomas, epidermoid cysts, and Mullerian cysts [1]. The diagnosis of these masses is challenging since CT findings can overlap, and aspiration of the fluid can fail to identify the type of cells that line the cyst [2]. As such, the final diagnosis typically occurs following surgical resection of these masses.

The rarity, as well as the similarity in terminology and clinical features, of pancreatic mucinous cystic neoplasms (MCNs), primary retroperitoneal MCNs, and extraovarian MCNs has made these entities difficult to characterize. MCNs are rare neoplastic lesions that are defined as having mucin-producing neoplastic epithelial cells with characteristic ovarian-type subepithelial stroma [3]. MCNs can be found on the ovaries and in extraovarian tissue such as the pancreas, mesentery, and retroperitoneum [4–6]. To date, only 28 cases have been described in the literature of MCNs arising in the mesentery or the retroperitoneum [4–8]. These neoplasms have almost been exclusively reported in women and on average present during the fourth decade [6, 9]. To the best of our knowledge, this is the first case reported of a MCN arising in the retroperitoneum related to a side branch intraductal papillary mucinous neoplasm (IPMN) of the pancreas.

## 2. Case Report

A 70-year-old female with a past medical history of Type 1 diabetes mellitus (T1DM), interstitial lung disease, hypothyroidism, and rheumatoid arthritis (RA) was referred to our urology clinic for a retroperitoneal mass. The patient's mass was first identified on a pelvic ultrasound (US). Physical exam at the time was normal except for some fullness noted near the left ovary. Pelvic US showed a thickened endometrium and a large cystic lesion with floating echogenic debris in the left lower quadrant measuring 16 × 11.1 × 8.7 cm. The radiologist's impression of the US noted that the cystic lesion was unlikely to be ovarian in origin and recommended a CT of the abdomen and pelvis to further evaluate the lesion.

**Figure 1:**
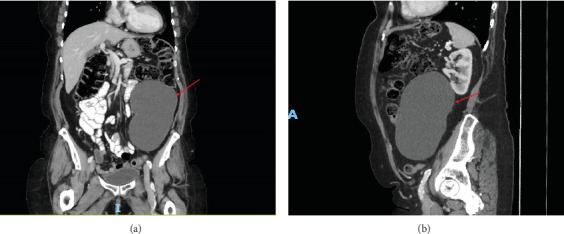
(a) Coronal view and (b) sagittal view of contrast-enhanced CT of the abdomen and pelvis showing a 9.4 × 16.1 × 10.6 cm cystic lesion within the left lower quadrant causing mild local mass effect.

**Figure 2:**
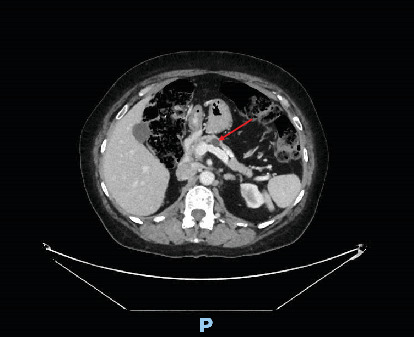
Contrast-enhanced CT of the abdomen and pelvis showing a 1.2 cm cystic lesion within the body of the pancreas.

This CT is shown in Figures 1 and 2 and revealed a large cystic lesion with few thin septations within the left lower quadrant measuring 9.4 × 16.1 × 10.6 cm and potentially arising from the mesentery. A 1.2 cm cystic lesion within the body of the pancreas was also identified and was consistent with a side branch IPMN. The differential diagnosis for the retroperitoneal lesion at this time was either a mesenteric cyst or a lymphangioma.

**Figure 3:**
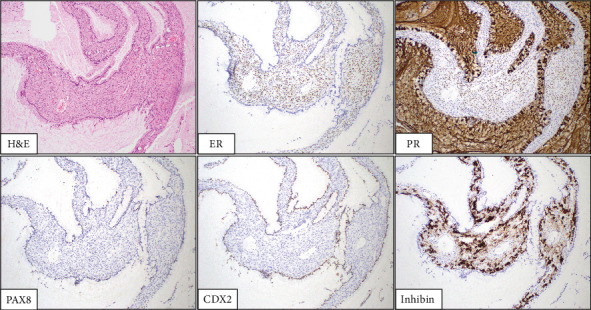
100× magnification. The H&E shows pink, ovarian-type stroma lined by columnar, mucinous-type epithelium with apical mucin. Immunostains for ER, PR, and inhibin highlight the ovarian-type stromal cells. The mucinous epithelium is highlighted by CDX2, and PAX8 highlights rare epithelial cells.

**Figure 4:**
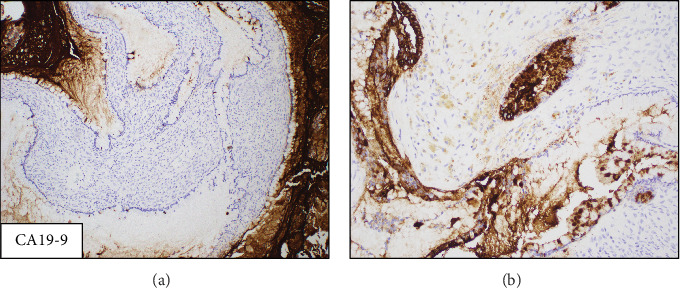
(a)100× and (b) 200× magnifications. CA19-9 shows variable expression in the epithelial cells, ranging from no expression to diffuse.

The patient's worsening constipation refractory to medication was attributed to the mass effect from this lesion, and the mass was successfully removed in an open extraperitoneal fashion without complications. Intraoperatively, the mass was fluid-filled, easily dissected, and not adherent to any structure. Histology slides are shown in Figures 3 and 4. Histopathologic examination demonstrated large cysts lined by a single layer of mucinous-type epithelial cells with underlying ovarian-type stroma. No cytologic atypia or significant mitotic activity was identified. By immunohistochemistry, the epithelial cells were labeled for carbohydrate antigen 19-9 (CA 19-9) and caudal-related homeobox transcription Factor 2 (CDX2) with rare paired-box Gene 8 (PAX8) positivity. The ovarian-type stromal cells were labeled for the estrogen receptor (ER), progesterone receptor (PR), and inhibin. The overall findings supported the diagnosis of a MCN with ovarian-type stroma.

The patient was seen 2 weeks later for her postoperative visit and reported that her constipation had significantly improved. The patient was subsequently referred to our Pancreatic Cyst Clinic where interval imaging was recommended given the absence of high-risk features. Imaging 3 months after surgery found no evidence of residual or recurrent disease and no change in the IPMN. The patient is currently scheduled for follow-up and reimaging annually for 5 years with us and twice annually with our Pancreatic Cyst Clinic.

## 3. Discussion

Distinguishing the pathophysiology and histology between pancreatic and retroperitoneal MCNs is challenging, especially considering their rarity, but our patient presents even more uncertainty as her case has components of both pancreatic and primary retroperitoneal MCNs. With the increase in use of abdominal imaging, the majority of primary retroperitoneal MCNs have been found incidentally. However, four cases have presented with either right lower quadrant pain or pelvic pain [6, 8]. While our patient's mass was found incidentally on imaging, she had concurrent symptoms which may or may not have been related. To our knowledge, this is the first report of an extra pancreatic retroperitoneal MCN associated with an IPMN of the pancreas.

Historically, retroperitoneal and mesentery MCNs were labeled as mucinous cystadenomas and thought to be related to their ovarian counterparts. As such, patient's ovaries were often examined and at times resected [4]. Of the two separate case series, only one patient has had a concomitant ovarian lesion. To the authors' knowledge, this is the first case of a retroperitoneal MCN presenting with a concomitant pancreatic cyst. It remains unclear whether retroperitoneal MCNs are disease processes related to their pancreatic or ovarian counterparts or unique entities of their own. Pancreatic MCNs are the second most common cystic pancreatic lesion with the greatest prevalence in middle-aged women [10]. Over 90% of MCNs are found in the body or tail of the pancreas, with the remainder presenting in the pancreatic head [9]. Primary retroperitoneal MCNs typically present at larger sizes, ranging from approximately 10 to 20 cm in greatest diameter, and are usually found in the lateral retroperitoneal space, as in our patient [11]. This larger size at presentation, compared to ovarian and pancreatic masses, is due to the location of the mass allowing for meaningful growth before symptoms develop.

It is important to note that not every case report of retroperitoneal MCNs presents with the characteristic ovarian-like stroma, suggesting the possibility of separate entities being reported as primary retroperitoneal MCNs in the literature [6, 8]. A recent case series by Van Treeck et al. looked at seven separate MCNs arising from the abdominal mesentery or the retroperitoneum. They found that in all seven cases, the epithelium was positive for Keratin 7 and Keratin 19, and the ovarian-like stroma was positive for ER, PR, and steroidogenic factor-1 (SF-1) [6]. The case of MCN at our institution was positive for CDX2 and PAX8 (rare cells), which is consistent with the one out of three and two out of three cases in the case series by Van Treeck et al.; Keratins 7 and 19 were not performed [6]. CDX2 is a commonly used marker to confirm a gastrointestinal origin, especially in the lower gastrointestinal tract, but can be expressed in any tumor along this tract or with an enteric histologic phenotype [12]. Its expression suggests that the MCN may have originated from the GI tract, although this finding alone is nonspecific.

To our knowledge, only one prior case has used CA 19-9 as a diagnostic tool to evaluate retroperitoneal MCNs. However, the case mentioned found that CA 19-9 serum levels were elevated in the patient and subsequently dropped after the removal of the mass [13]. Unique to our case is the use of CA 19-9 as an immunostain to evaluate the mass, which has not been reported in other studies. CA 19-9 is generally used in the setting of pancreatic malignancy, and its expression in this case suggests a similarity between pancreatic tissue and the tissue that makes up retroperitoneal MCNs [14]. Additionally, our specimen's ovarian-like stroma was also positive for ER and PR, as well as inhibin which was positive in five out of six cases in the case series by Van Treeck et al. [6]. To our knowledge, this is the first instance reported of a retroperitoneal MCN with positivity for CA 19-9. This finding can point to a possible diagnostic tool in the identification and classification of these rare masses but requires further research.

Differentiating primary retroperitoneal from pancreatic MCNs is of clinical significance as it is not clear whether the former carry the same malignant potential as their pancreatic counterparts. Pancreatic MCNs are generally considered to be benign lesions; however, a small subset of lesions can progress to invasive adenocarcinoma. This risk especially increases with age and size of the mass [15, 16]. Interestingly, despite our patient's older age and a mass size of 16 cm, the cyst lining lacked any carcinomatous cells thus suggesting a fully benign mucinous cystadenoma. Large pancreatic MCNs are usually managed with complete resection with no further postoperative management if the mass is noninvasive. If there is invasion, the 5-year survival drops significantly from 100% to 26% [3]. However, due to the rarity of primary retroperitoneal MCNs, no long-term follow-up data exists to definitively characterize survival.

Here, we present the first case of a primary retroperitoneal MCN presenting with a concomitant pancreatic cystic lesion. Because of the rarity of these lesions, the relationship between retroperitoneal MCNs, pancreatic MCNs, and ovarian MCNs remains unclear. However, the histologic similarities between these lesions suggest possible shared risk factors and pathogenesis. Additionally, the similarities between these lesions and changes in nomenclature over time have made it difficult to classify extraovarian MCNs as separate or related entities. Overall, more research is needed to clearly define the diagnostic characteristics of retroperitoneal MCNs. Additionally, the relationship between retroperitoneal MCNs and IPMN needs further research to further inform the long-term management and prognosis of these lesions.

## 4. Conclusion

Retroperitoneal MCNs are very rare cystic lesions most commonly presenting in women. Risk factors and predisposing conditions for retroperitoneal MCNs have not been well documented. While these lesions are considered potential precursors to malignant lesions in the pancreas, so far, surgical resection of retroperitoneal MCNs appears to be curative with no recurrence reported in the literature to date. Urologists should be aware of this uncommon retroperitoneal mass and its behavior as we are increasingly involved in the diagnosis and management of patients with these findings due to our familiarity with surgery in the space.

## Data Availability

The authors have nothing to report.
